# Is glucose-6-phosphate dehydrogenase deficiency associated with COVID-19 infection, severity, and death? A cohort study from the Brazilian Amazon

**DOI:** 10.1371/journal.pone.0331729

**Published:** 2025-12-23

**Authors:** Adila L. B. Dias, Joabi Rocha Nascimento, José Diego Brito-Sousa, Marco Aurelio Sartim, Kamilla Freitas da Silva, Alexandre Vilhena Silva-Neto, Gabriel dos Santos Mouta, Patricia Carvalho da Silva Balieiro, Djane Clarys Baia-da-Silva, Fernando Almeida-Val, Gisely Cardoso de Melo, Vanderson de Souza Sampaio, Marcus Lacerda, Wuelton Monteiro

**Affiliations:** 1 Fundação de Medicina Tropical Dr Heitor Vieira Dourado, Manaus, Brazil; 2 Escola Superior de Ciências da Saúde, Universidade do Estado do Amazonas, Manaus, Brazil; 3 Instituto de Pesquisas Leônidas & Maria Deane, Fundação Oswaldo Cruz, Manaus, Brazil; 4 Faculdade de Ciências Farmacêuticas, Universidade Federal do Amazonas, Manaus, Brazil; 5 Università degli Studi di Siena, Siena, Italy; 6 Universidade Nilton Lins, Manaus, Brazil; 7 Instituto Todos pela Saúde, São Paulo, Brazil; 8 University of Texas Medical Branch, University of Texas, Galveston, United States of America; 9 Duke Global Health Institute, Duke University, Durham, United States of America; Menzies School of Health Research, AUSTRALIA

## Abstract

**Background:**

Glucose-6-phosphate dehydrogenase deficiency (G6PDd) is a common genetic disorder that impairs the cellular antioxidant response and has been hypothesized as a potential risk factor for severe outcomes in viral infections, including COVID-19. However, clinical evidence remains limited, especially in regions with high G6PDd prevalence.

**Methods:**

We conducted a retrospective cohort study using secondary data from four health information systems from health facilities in the Brazilian Amazon (E-SUS Notifica, SIVEP-Gripe, SIM, and a G6PD enzyme activity database). The study population consisted of 3,955 male participants, including 206 with confirmed G6PDd. We used logistic regression to assess associations between G6PDd and COVID-19 infection, hospitalization, and death. Cox proportional hazards models and Kaplan-Meier curves were applied to evaluate time-to-event outcomes.

**Results:**

No statistically significant association was found between G6PDd and SARS-CoV-2 infection (OR = 1.15; 95% CI: 0.70–1.79; p = 0.6), hospitalization (OR = 1.13; 95% CI: 0.27–3.20; p = 0.8), or death (OR = 0.00; p > 0.9). Age was a significant risk factor for all outcomes, and individuals identified as Asian had a higher likelihood of infection (OR = 2.87; 95% CI: 1.57–5.29; p < 0.001).

**Conclusion:**

In this cohort from the Brazilian Amazon, G6PD deficiency was not associated with an increased risk of COVID-19 infection or severe outcomes. These findings emphasize the importance of considering ethnic and genetic diversity in epidemiological analyses and health policy planning, particularly in regions with high G6PDd prevalence.

## Introduction

The COVID-19 pandemic, caused by SARS-CoV-2, has had a major global impact since its emergence in December 2019 in Wuhan, China [1–3]. The World Health Organization (WHO) declared a Public Health Emergency of International Concern on January 30, 2020, and officially characterized it as a pandemic on March 11 of the same year [4,5]. In Brazil, the pandemic was particularly severe, with approximately 39.3 million confirmed cases and more than 700,000 deaths [6]. The state of Amazonas was one of the most affected regions, reporting 65,173 confirmed cases and 14,556 deaths, and facing a dramatic collapse in healthcare infrastructure, especially in Manaus, due to shortages of oxygen and hospital beds [7].

COVID-19 manifests with a broad clinical spectrum, from asymptomatic infection to severe illness, including pneumonia, respiratory failure, and multiorgan dysfunction [8–10]. Approximately 32% of SARS-CoV-2 infections are asymptomatic, and 20% of people remain symptom-free throughout the infection [11,12]. Among symptomatic individuals, 80% presented mild or moderate illness, 15% developed severe disease, and 5% progressed to critical illness with severe complications [11,13]. Several demographic and clinical factors influence disease severity, notably advanced age and the presence of preexisting comorbidities. Among these factors, genetic variations may also play a role during COVID infection, and some polymorphisms have been found to protect or outcomes.

G6PD is a key enzyme in the pentose phosphate pathway and is used to protect cells from oxidative stress [14,15]. G6PD deficiency, affecting over 400 million people worldwide, is characterized by reduced enzymatic activity and increased vulnerability to oxidative damage and hemolysis. This condition is a common enzymopathy linked to the Xq28 locus of the X chromosome. Although it is classified as recessive, both homozygous and heterozygous individuals may exhibit symptoms [16,17]. In Brazil, the estimated prevalence is around 5%, primarily due to the African A−variant [18]. Preclinical and clinical studies have shown that G6PDd, whether congenital or acquired, may be associated with increased or decreased vulnerability to SARS-CoV-2 infection [19–21]. While studies have shown that G6PD deficiency can also exacerbate specific aspects of COVID-19, such as respiratory dysfunction, anemia, and hepatic impairment [22], others found no association between G6PDd and mortality or need for ventilatory support [23]. Therefore, the clinical relationship between G6PD deficiency and COVID-19 severity is controversial and requires further investigation.

Health databases such as E-SUS Notifica, SIVEP-Gripe, and the Mortality Information System (SIM) are valuable tools for epidemiological surveillance and research in Brazil. These systems allow for the collection and integration of large-scale clinical and demographic data, contributing to evidence-based decision-making during pandemics [24–27].

This study aimed to investigate the association between G6PD deficiency and the incidence, hospitalization, and mortality due to COVID-19 in a male cohort from the Brazilian Amazon. Given the regional prevalence of G6PDd and the socioeconomic and ethnic diversity of the population, understanding this relationship may inform clinical management and public health strategies.

## Materials and methods

### Study design and setting

**Fig 1:**
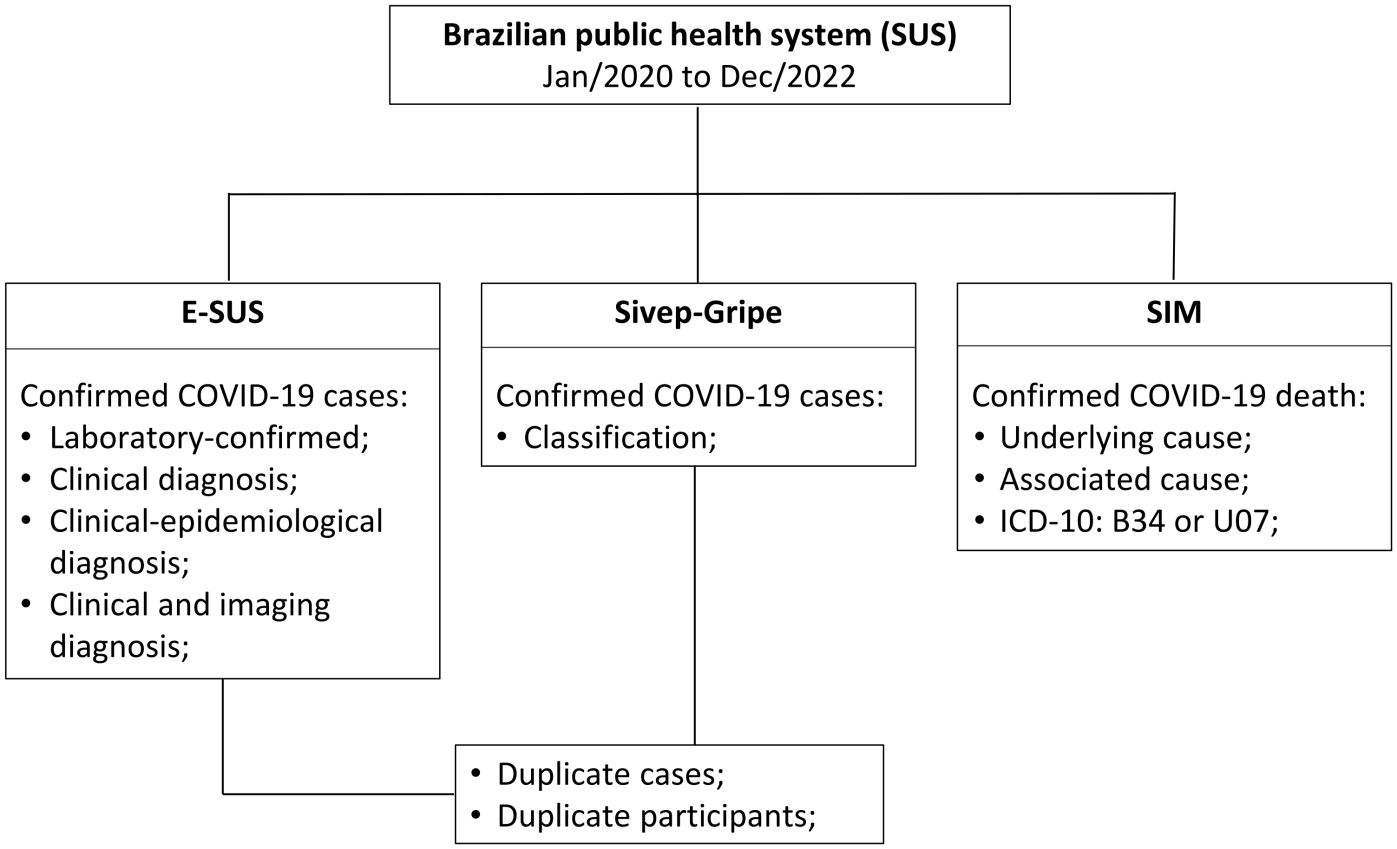
Flowchart describing data collection from the Brazilian Unified Health System (SUS) information systems.

This was a retrospective cohort study (January 1, 2020 – December 31, 2022), conducted using clinical, demographic, epidemiological, and outcome data from Brazilian national databases: **e-SUS Notifica**, which records confirmed COVID-19 cases; **SIVEP-Gripe**, which includes information on COVID-19-related hospitalizations; and **SIM** (Mortality Information System), which contains records of deaths related to COVID-19 **(**Fig 1**)**. A database containing G6PD enzyme activity data from patients was compiled from three separate studies that evaluated G6PD status in distinct populations. Identification data from participants were accessed to link all the datasets and were deleted as soon as the final dataset was built. All the datasets were accessed on July 1, 2023. For this study, only male individuals were included in the analyses. The methods used to identify G6PD deficiency are described in the original papers [28–32], properly approved by research ethic committees (CAAE: 92012818.1.0000.0005, CAAE 16867319.6.0000.0008, CAEE: 8307814.7.0000.0005). This study was approved by the research ethics committee (CAAE: 45714621.2.0000.0005), which waived the requirement for informed consent. Data were obtained from different sources, including public health information systems from the state of Amazonas, Brazil, heavily affected by COVID-19 [7].

### Data processing: deduplication and record linkage

The databases did not contain unique identifiers that would allow deterministic linkage. Therefore, they underwent a standardization process that included the removal of special characters, accents, and prepositions from participant names, as well as the formatting of birth dates. When birth dates were unavailable, recorded age was used for adjustment.

To remove redundant records, probabilistic deduplication was performed using the RecordLinkage package in R. The command RLBigDataDedup was used with the variables “Participant Name,” “Mother’s Name,” and “Date of Birth”; age was used as a substitute when the date of birth was missing. Next, probabilistic linkage between databases was performed to associate G6PD deficiency data with COVID-19, hospitalization, and death records. The linkage used the R programming language (RStudio 4.2.1) and the RecordLinkage library, employing the Levenshtein phonetic algorithm and blocking by sex. Identified pairs were manually reviewed by experts, who determined the match cutoff score.

### Data analysis

The main explanatory variable was G6PD deficiency. In this study, participants with intermediate enzymatic activity were classified as having normal levels. The outcomes analyzed were confirmed COVID-19 infection, hospitalization, ICU admission, and death. Confirmed cases were identified in the SIVEP-Gripe and E-SUS Notifica databases. Hospitalizations were identified in SIVEP-Gripe, and deaths were included if COVID-19 was listed as the underlying or associated cause in the SIM.

Logistic regression was used to analyze associations between G6PD deficiency, ethnicity, age, and the outcomes (p < 0.05). Pearson’s chi-squared test was used for categorical variables, and the Wilcoxon test for continuous variables. Additionally, survival analysis was conducted to assess the association between G6PD deficiency and SARS-CoV-2 infection, time to hospitalization, ICU admission, and death, using the Cox proportional hazards model. Kaplan–Meier curves were used to estimate survival probabilities, and the log-rank test was used to compare groups.

All analyses were performed in R (version 4.4) and RStudio (2024.09.01), using the tidyverse, survival, and survminer packages. Graphs included 95% confidence intervals, and a 5% significance level was adopted.

## Results

**Fig 2:**
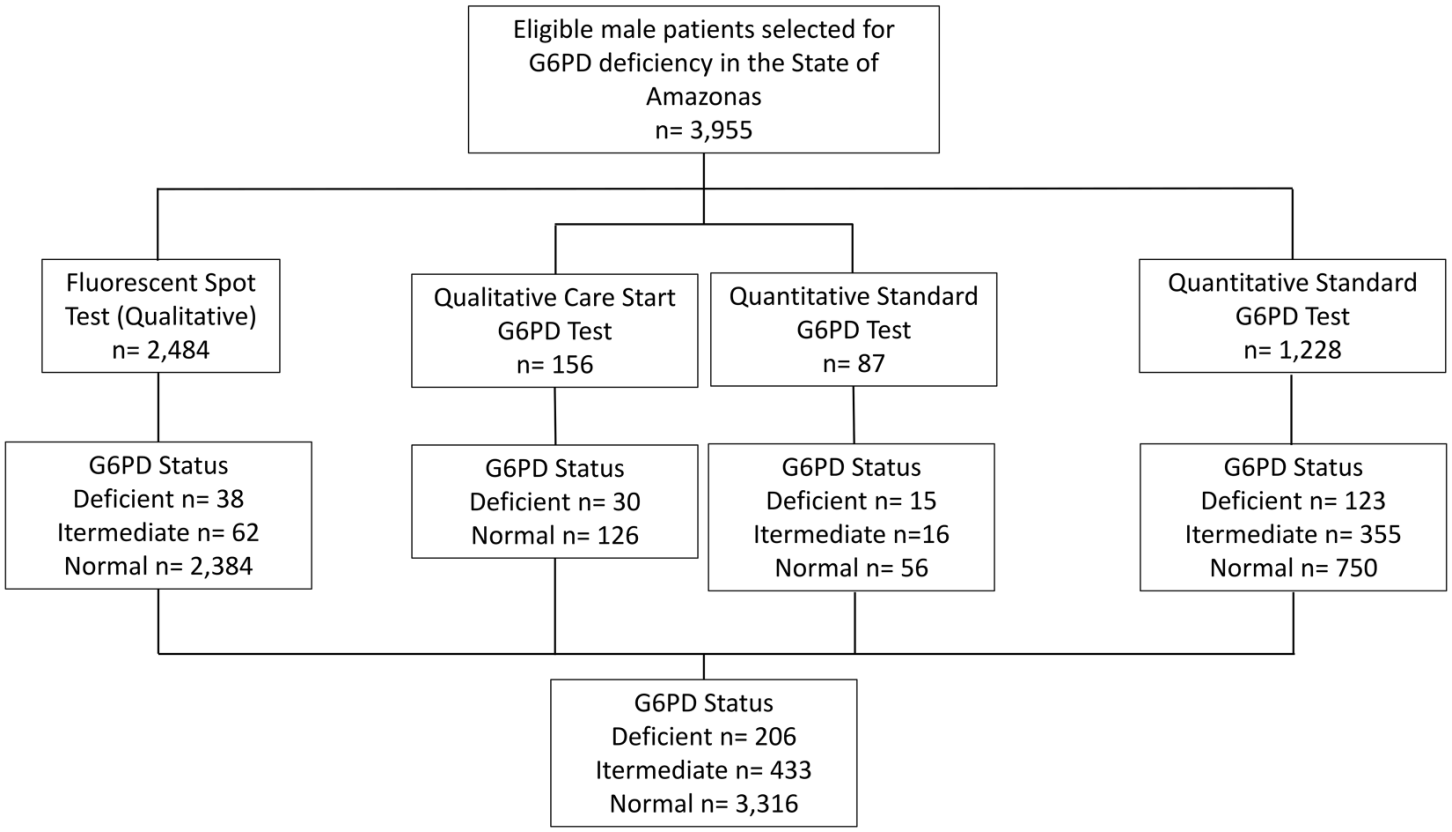
Flowchart describing the samples from the studies that compose the G6PD deficiency database.

The study sample included 3,955 male participants, of whom 206 (5.2%) had G6PD deficiency (Fig 2). The demographic characteristics of participants are presented in **Table 1**. The mean age was very symmetrical among participant groups, with 35 years for both the total and Normal G6PD groups, and 34 years for the G6PD-deficient group. Regarding race, participants who self-reported admixed ethnicity consisted of the majority in all groups (over 72%); however, a noticeable distinction was observed in G6PD deficiency among Black (7.77%) and Asian (1.46%) participants.

**Table 1:** Demographic and clinical characteristics of study participants.

Characteristics	Total N = 3,955	Normal	G6PD deficient
		N = 3,749	N = 206
**Age, mean (SD)**	35 (17)	35 (17)	34 (18)
**Ethnicity, n (%)**			
White	259 (6.55%)	245 (6.54%)	14 (6.80%)
Black	612 (15.47%)	596 (15.90%)	16 (7.77%)
Asian	125 (3.16%)	122 (3.25%)	3 (1.46%)
Admixed	2,899 (73.30%)	2,729 (72.79%)	170 (82.52%)
Indigenous	60 (1.52%)	57 (1.52%)	3 (1.46%)

In the multivariate analysis, no statistically significant association was observed between G6PD deficiency and SARS-CoV-2 infection (OR = 1.15; 95% CI: 0.70–1.79; p = 0.6) (**Table 2**). Age was significantly associated with infection (OR = 1.02; p < 0.001), as was Asian (OR = 2.87; 95% CI: 1.57–5.29; p < 0.001).

**Table 2:** Association between G6PD deficiency, demographic characteristics, and COVID-19 infection.

	Descriptive				Univariate Regression			Multivariate Regression		
Characteristic	Total N = 3,955	No Covid infection N = 3,579	Covid infection N = 376	p-value^1^	OR^2^	95% CI^2^	p-value	OR^2^	95% CI^2^	p-value
**G6PD deficient, N (%)**	206 (5.21%)	185 (5.17%)	21 (5.59%)	0.7	1.09	0.66; 1.69	0.7	1.15	0.70; 1.79	0.6
**Age, Average (SD)**	34.8 (16.9)	34.3 (17.0)	39.4 (15.3)	**<0.001**	1.02	1.01; 1.02	**<0.001**	1.02	1.01; 1.02	**<0.001**
**Race, n/N (%)**				**<0.001**						
White	259 (6.55%)	236 (6.59%)	23 (6.12%)		—	—		—	—	
Black	612 (15.47%)	553 (15.45%)	59 (15.69%)		1.09	0.67; 1.85	0.7	1.01	0.61; 1.71	>0.9
Asian	125 (3.16%)	97 (2.71%)	28 (7.45%)		2.96	1.63; 5.44	**<0.001**	2.87	1.57; 5.29	**<0.001**
Admixed	2,899 (73.30%)	2,643 (73.85%)	256 (68.09%)		0.99	0.65; 1.59	>0.9	0.98	0.64; 1.57	>0.9
Indigenous	60 (1.52%)	50 (1.40%)	10 (2.66%)		2.05	0.89; 4.48	0.079	2.04	0.88; 4.46	0.083

^1^Pearson’s Chi-squared test; Wilcoxon rank sum test

^2^OR = Odds Ratio, CI = Confidence Interval

No association was found between G6PD deficiency and hospitalization (OR = 1.13; 95% CI: 0.27–3.20; p = 0.8) or death (all 16 deaths were in the non-deficient group), even after adjustment for age and race. Age was strongly associated with both outcomes: for hospitalization, OR = 1.06 (p < 0.001); for death, OR = 1.06 (p < 0.001). No racial group was significantly associated with hospitalization or mortality in the adjusted models (Tables 3 and 4).

**Table 3:** Association between G6PD deficiency, demographic characteristics, and hospitalization due to COVID-19.

	Descriptive				Univariate Regression			Multivariate Regression		
Characteristic	Total N = 3,955	Not hospitalized N = 3.903	Hospitalized N = 52	p-value^1^	OR^2^	95% CI^2^	p-value	OR^2^	95% CI^2^	p-value
**G6PD deficient, N (%)**	206 (5.21%)	203 (5.20%)	3 (5.77%)	0.8	1.12	0.27; 3.07	0.9	1.13	0.27;3.20	0.8
**Age, mean (SD)**	34.8 (16.9)	34.6 (16.8)	51.6 (17.9)	**<0.001**	1.06	1.04; 1.08	**<0.001**	1.06	1.04;1.08	**<0.001**
**Race, N (%)**				0.3						
White	259 (6.55%)	255 (6.53%)	4 (7.69%)		—	—		—	—	
Black	612 (15.47%)	604 (15.48%)	8 (15.38%)		0.84	0.26; 3.19	0.8	0.65	0.20;2.50	0.5
Asian	125 (3.16%)	121 (3.10%)	4 (7.69%)		2.11	0.49; 9.05	0.3	1.97	0.45;8.65	0.4
Admixed	2,899 (73.30%)	2,864 (73.38%)	35 (67.31%)		0.78	0.31; 2.62	0.6	0.78	0.30;2.64	0.6
Indigenous	60 (1.52%)	59 (1.51%)	1 (1.92%)		1.08	0.05; 7.47	>0.9	1.21	0.06;8.69	0.9

^1^Fisher’s exact test; Wilcoxon rank sum test

^2^OR = Odds Ratio, CI = Confidence Interval

**Table 4:** Association between G6PD deficiency, demographic characteristics, and death from COVID-19.

	Descriptive				Univariate Regression			Multivariate Regression		
Characteristic	Total N = 3,955	No death N = 3.939	Death N = 16	p-value^1^	OR^2^	95% CI^2^	p-value	OR^2^	95% CI^2^	p-value
**G6PD deficient, N (%)**	206 (5.21%)	206 (5.23%)	0 (0.00%)	>0.9	0.00		>0.9	0.00		>0.9
**Age, mean (SD)**	34.8 (16.9)	34.8 (16.9)	52.0 (17.4)	**<0.001**	1.06	1.03; 1.09	**<0.001**	1.06	1.03; 1.09	**<0.001**
**Race, N (%)**				0.061						
White	259 (6.55%)	259 (6.58%)	0 (0.00%)		—	—		—	—	
Black	612 (15.47%)	606 (15.38%)	6 (37.50%)		Inf^4^	0.00; NA^3^	>0.9	Inf^4^	0.00; NA^3^	>0.9
Asian	125 (3.16%)	125 (3.17%)	0 (0.00%)		1.00	0.00; Inf^4^	>0.9	0.85	0.00; Inf^4^	>0.9
Admixed	2,899 (73.30%)	2,890 (73.37%)	9 (56.25%)		Inf^4^	0.00; NA^3^	>0.9	Inf^4^	0.00; NA^3^	>0.9
Indigenous	60 (1.52%)	59 (1.50%)	1 (6.25%)		Inf^4^	0.00; NA^3^	>0.9	Inf^4^	0.00; NA^3^	>0.9

^1^Fisher’s exact test; Wilcoxon rank sum test

^2^OR = Odds Ratio, CI = Confidence Interval

^3^NA = Not Applicable

^4^Inf = a very large numeric value

**Fig 3:**
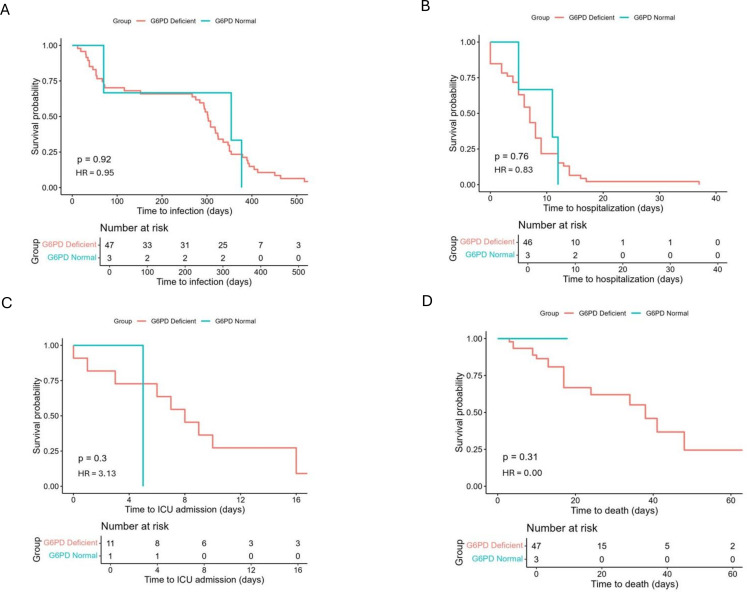
Kaplan–Meier curve comparing the probability of infection over time by G6PD status; A) Time to infection; B) Time to hospitalization; C) Time to ICU admission; D) Time to death in COVID-19 patients by G6PD status.

Kaplan–Meier curves (Fig 3A–3D) were used to evaluate time to infection, hospitalization, ICU admission, and death by G6PD status. No significant differences were observed in any of the time-to-event outcomes, further supporting the absence of association between G6PD deficiency and adverse COVID-19 progression.

In addition to the main analysis, we performed two sensitivity analyses to assess the robustness of our findings. The first included only participants who had been screened for infectious diseases at the time of G6PD testing [31], aiming to minimize potential bias related to transient alterations in enzymatic activity. The second involved participants matched by G6PD status and age to account for potential confounding (**S1****–****S6 Tables**). In both subgroups, the results remained consistent with those of the full cohort, showing no significant associations between G6PD deficiency and COVID-19 outcomes. These findings support the internal validity of the study and suggest that the observed lack of association is unlikely to be due to sample composition or measurement bias.

## Discussion

This retrospective cohort study evaluated the association between G6PDd and COVID-19 outcomes – infection, hospitalization, and mortality – in a male population whose enzymatic activity had been measured before the pandemic. Although early hypotheses suggested that G6PDd could worsen the clinical course of coronavirus infections due to its role in cellular oxidative stress responses [33,34], our findings did not demonstrate a statistically significant association between G6PDd and any of the evaluated outcomes in this population.

Specifically, G6PD-deficient (G6PDd) individuals were not at increased risk of SARS-CoV-2 infection, hospitalization, or death. These results are consistent with those of de Almeida et al. [23], who evaluated G6PD enzymatic activity and COVID-19 severity in a hospitalized cohort in the same region. That study also found no association between G6PDd and mortality or need for ventilatory support, although it did report a longer hospital stay for G6PDd patients. Together, these findings suggest that while G6PDd may influence some clinical aspects of care, it does not appear to directly impact the most severe outcomes of COVID-19.

A study conducted among United States veterans found an increased risk of severe COVID-19 in G6PD-deficient subgroups, particularly among Black veterans under 65 and White veterans aged 65 and older, suggesting that the relationship between G6PDd and disease severity may be modulated by age and ethnicity [20]. These data imply that G6PDd alone may not be a determinant of clinical severity but should be considered alongside other modifying factors in future analyses.

One potential explanation for the lack of association observed in our study relates to the predominant genetic variant of G6PD in the Brazilian Amazon, the G6PD*A− variant, and its repercussion on cell oxidative stress. This variant, classified as a Class III mutation, is associated with moderate enzymatic deficiency and partial preservation of redox capacity [31]. Such a mild phenotype may not be sufficient to impair antioxidant responses to the extent necessary to influence the clinical course of COVID-19. In contrast, Class I and II variants, which are more prevalent in the Mediterranean, Middle East, and parts of Asia, result in more severe enzymatic impairment and have been associated with increased vulnerability to infectious diseases. Studies suggest that it is the degree of enzymatic deficiency, rather than the mere presence of a G6PD mutation, that critically influences susceptibility to adverse outcomes, especially under conditions of heightened oxidative stress such as severe viral infections [35,36]. Additionally, the small number of severe events (hospitalizations and deaths) among G6PD-deficient individuals in our cohort may have limited the statistical power to detect meaningful associations. This further underscores the need for larger multicenter studies capable of stratifying participants by variant class and clinical severity.

The survival analyses of time-to-event outcomes (hospitalization, ICU admission, and death) also revealed no significant differences between G6PD-deficient and non-deficient groups. This temporal similarity supports the regression results and suggests that G6PDd does not impact disease progression. In contrast, age consistently emerged as a strong risk factor across all outcomes measured. This aligns with a broad body of literature demonstrating that advanced age is associated with worse COVID-19 outcomes regardless of other risk factors [37–40].

Another relevant finding was the significant association between Asian ethnicity and an increased risk of SARS-CoV-2 infection. This observation is supported by studies such as that by Sze et al. [41], which found heightened COVID-19 susceptibility among individuals of Asian descent in the UK, even after adjusting for socioeconomic factors. While racial analyses in Brazil traditionally focus on disparities affecting Black and Admixed populations [42], our findings underscore the importance of expanding vulnerability assessments to include other ethnic groups, particularly in demographically diverse regions such as the Amazon.

The increased risk observed among Asian-descendant participants in our study mirrors available evidence, where higher infection and hospitalization rates were noted among Asian populations [43]. These disparities are likely multifactorial, potentially reflecting occupational exposure, population density, genetic predisposition, or differential access to healthcare. However, in the Brazilian context, such associations remain underexplored. Further research is warranted to better understand the determinants of risk in Asian-descendant groups and ensure their adequate representation in public health surveillance and policy development.

The lack of association between G6PDd and severe COVID-19 may also reflect phenotypic variability [17]. Clinical expression of G6PDd likely depends on interactions with contextual oxidative stressors such as certain drugs (e.g., sulfonamides and primaquine) or coinfections, which may not have been present or prevalent in our cohort [44].

Our sensitivity analyses support the robustness of the findings. These analyses included participants who tested negative for malaria and were afebrile at the time of G6PD testing, aiming to avoid enzymatic activity modulation by acute infections [45,46]. Because phenotypic tests measure enzymatic activity rather than genotype, the exclusion of participants with active infections was intended to reduce potential misclassification. Additionally, we performed age-matched subgroup analyses to mitigate sample distribution bias, given the 5.2% G6PDd prevalence. The consistency of these results with the main findings further reinforces the internal validity of the study.

This study has some limitations, including the lack of data on viral load, comorbidities, and vaccination status; the potential underreporting of mild or asymptomatic cases; the restriction to male participants, due to the methodology used to identify complete G6PD deficiency; and the fact that G6PD testing was not performed during the pandemic. One notable limitation is the absence of granular clinical data, such as the need for mechanical ventilation or laboratory markers of severity, which have been used in other studies to capture intermediate clinical outcomes. Although we evaluated hospitalization and mortality, we were unable to assess whether G6PD deficiency influenced other severe outcomes. Moreover, the relatively small number of hospitalizations and deaths among G6PD-deficient individuals reduced the statistical power to detect associations with severe COVID-19 outcomes. Finally, the absence of molecular G6PD genotyping limits the ability to evaluate variant-specific effects, particularly for rare Class I and II mutations that may confer higher clinical risk. It should be noted that the African variant, classified as a Class III variant, predominates in the study area [15], which may have explained our results showing a low impact of the deficiency on the outcomes studied.

## Conclusion

In this Amazonian cohort, G6PD deficiency was not associated with increased risk of COVID-19 infection, hospitalization, ICU admission, or death. These findings likely reflect the predominance of Class III G6PD variants in the region and should be interpreted accordingly. Age remained the strongest predictor of adverse outcomes.

## Supporting information

S1 Table Descriptive and regression sensitivity analysis of COVID-19 incidence in individuals with and without G6PD deficiency. Only participants who had been screened for infectious diseases at the time of G6PD testing were included.
(DOCX)

S2 Table Descriptive and regression sensitivity analysis of COVID-19 hospitalization in individuals with and without G6PD deficiency. Only participants who had been screened for infectious diseases at the time of G6PD testing were included.
(DOCX)

S3 Table Descriptive and regression sensitivity analysis of death from COVID-19 in individuals with and without G6PD deficiency. Only participants who had been screened for infectious diseases at the time of G6PD testing were included.
(DOCX)

S4 Table Descriptive and regression sensitivity analysis of COVID-19 infection in a subsample matched by age. (DOCX)

S5 Table Descriptive and regression sensitivity analysis of hospitalization due to COVID-19 in a sample matched by age. (DOCX)

S6 Table Descriptive and regression sensitivity analysis of death from COVID-19 in a sample matched by age. (DOCX)

## References

[1] Wang C, Horby PW, Hayden FG, Gao GF. A novel coronavirus outbreak of global health concern. Lancet. 2020;395(10223):470–3. doi: 10.1016/S0140-6736(20)30185-9 31986257 PMC7135038

[2] Wu Z, McGoogan JM. Characteristics of and Important Lessons From the Coronavirus Disease 2019 (COVID-19) Outbreak in China: Summary of a Report of 72 314 Cases From the Chinese Center for Disease Control and Prevention. JAMA. 2020;323(13):1239–42. doi: 10.1001/jama.2020.2648 32091533

[3] World Health Organization. WHO-convened global study of origins of SARS-CoV-2: China part. Geneva: World Health Organization. 2021. https://www.who.int/publications/i/item/who-convened-global-study-of-origins-of-sars-cov-2-china-part

[4] Organização Pan-Americana da Saúde. Histórico da emergência internacional de COVID-19. OPAS/OMS.

[5] Umakanthan S, Sahu P, Ranade AV, Bukelo MM, Rao JS, Abrahao-Machado LF, et al. Origin, transmission, diagnosis and management of coronavirus disease 2019 (COVID-19). Postgraduate Medical Journal. 2020;96(1142):753–8. doi: 10.1136/postgradmedj-2020-138234 32563999 PMC10016932

[6] Ministério daSaúde Brasil. Covid-19 - Casos e Óbitos. [cited 7 Jul 2025]. Available: https://infoms.saude.gov.br/extensions/covid-19_html/covid-19_html.html

[7] Fundação de Vigilância em Saúde do Amazonas. Monitoramento da COVID‑19 no Amazonas. Portal FVS-RCP/AM. https://www.fvs.am.gov.br/indicadorSalaSituacao_view/60/2. Accessed 2025 July 7.

[8] Maggi E, Canonica GW, Moretta L. COVID-19: Unanswered questions on immune response and pathogenesis. J Allergy Clin Immunol. 2020;146(1):18–22. doi: 10.1016/j.jaci.2020.05.001 32389590 PMC7205667

[9] Jin Y, Yang H, Ji W, Wu W, Chen S, Zhang W, et al. Virology, Epidemiology, Pathogenesis, and Control of COVID-19. Viruses. 2020;12(4):372. doi: 10.3390/v12040372 32230900 PMC7232198

[10] Huang C, Wang Y, Li X, Ren L, Zhao J, Hu Y, et al. Clinical features of patients infected with 2019 novel coronavirus in Wuhan, China. The Lancet. 2020;395(10223):497–506. doi: 10.1016/s0140-6736(20)30183-5 PMC7159299 31986264

[11] Clinical management of COVID-19: Living guideline. https://www.who.int/publications/i/item/WHO-2019-nCoV-clinical-2023.2. 2023. Accessed 2025 July 9.

[12] Shang W, Kang L, Cao G, Wang Y, Gao P, Liu J, et al. Percentage of Asymptomatic Infections among SARS-CoV-2 Omicron Variant-Positive Individuals: A Systematic Review and Meta-Analysis. Vaccines. 2022;10(7):1049. doi: 10.3390/vaccines10071049 35891214 PMC9321237

[13] CDC Weekly C. The Epidemiological Characteristics of an Outbreak of 2019 Novel Coronavirus Diseases (COVID-19) — China, 2020. China CDC Weekly. 2020;2(8):113–22. doi: 10.46234/ccdcw2020.032 34594836 PMC8392929

[14] Stincone A, Prigione A, Cramer T, Wamelink MMC, Campbell K, Cheung E, et al. The return of metabolism: biochemistry and physiology of the pentose phosphate pathway. Biol Rev Camb Philos Soc. 2015;90(3):927–63. doi: 10.1111/brv.12140 25243985 PMC4470864

[15] Howes RE, Battle KE, Satyagraha AW, Baird JK, Hay SI. G6PD Deficiency. Advances in Parasitology. Elsevier. 2013:133–201. doi: 10.1016/b978-0-12-407826-0.00004-7 23384623

[16] Mehta A, Mason PJ, Vulliamy TJ. Glucose-6-phosphate dehydrogenase deficiency. Baillieres Best Pract Res Clin Haematol. 2000;13(1):21–38. doi: 10.1053/beha.1999.0055 10916676

[17] Nkhoma ET, Poole C, Vannappagari V, Hall SA, Beutler E. The global prevalence of glucose-6-phosphate dehydrogenase deficiency: a systematic review and meta-analysis. Blood Cells Mol Dis. 2009;42(3):267–78. doi: 10.1016/j.bcmd.2008.12.005 19233695

[18] Dombrowski JG, Souza RM, Curry J, Hinton L, Silva NRM, Grignard L, et al. G6PD deficiency alleles in a malaria-endemic region in the Western Brazilian Amazon. Malar J. 2017;16(1):253. doi: 10.1186/s12936-017-1889-6 28619120 PMC5471696

[19] Al-Abdi S, Al-Aamri M. G6PD deficiency in the COVID-19 pandemic: Ghost within Ghost. Hematol Oncol Stem Cell Ther. 2021;14(1):84–5. doi: 10.1016/j.hemonc.2020.04.002 32325028 PMC7166036

[20] Elsea SH, Razjouyan J, Lee KM, Lynch JA, Martini S, Pandit LM. Association of Glucose-6-Phosphate Dehydrogenase Deficiency With Outcomes in US Veterans With COVID-19. JAMA Netw Open. 2023;6(3):e235626. doi: 10.1001/jamanetworkopen.2023.5626 36988953 PMC10061239

[21] Vick DJ. Evaluation of glucose-6-phosphate dehydrogenase (G6PD) status in US military and VA patients with COVID-19 infection. BMJ Mil Health. 2021;167(2):144. doi: 10.1136/bmjmilitary-2020-001706 33243769

[22] Al Shbailat SA, Habash MH, Tarabsheh BO, Al Ammouri ZM, Suleiman R, Jaradat JH, et al. COVID-19 Severity and Associated Laboratory Tests Abnormalities in Glucose-6-Phosphate Dehydrogenase Deficiency Patients: A Systematic Review and Meta-Analysis. Oman Med J. 2025. doi: 10.5001/omj.2026.40

[23] de Almeida Rodrigues MG, Monteiro WM, de Melo GC, Dias ÁLB, Sartim MA, Xavier MS, et al. Associations between COVID-19 and Glucose-6-Phosphate Dehydrogenase Activity in Brazil. Am J Trop Med Hyg. 2024;110(6):1191–7. doi: 10.4269/ajtmh.23-0148 38593787 PMC11154054

[24] Ministério da Saúde Brasil, Fundação Nacional de Saúde. Manual de procedimentos do sistema de informações sobre mortalidade. Brasilia. 2001. http://bvsms.saude.gov.br/bvs/publicacoes/funasa/sis_mortalidade.pdf

[25] Coelho Neto GC, Andreazza R, Chioro A. Integration among national health information systems in Brazil: the case of e-SUS Primary Care. Rev Saude Publica. 2021;55:93. doi: 10.11606/s1518-8787.2021055002931 34878089 PMC8659614

[26] Guedes R, Dutra GJ, Machado C, Palma MA. Evaluation of data on deaths due to COVID-19 from the databases of the Civil Registry (RC-Arpen), SIVEP-Gripe, and SIM in Brazil in 2020. Cad Saude Publica. 2023;39(3):e00077222. doi: 10.1590/0102-311XPT077222 37018774

[27] Reis-Santos B. Health Information Systems: how much progress are we making?. Epidemiol Serv Saude. 2023;32(2):e2022433. doi: 10.1590/S2237-96222023000200001 37610937 PMC10443441

[28] Brito-Sousa JD, Murta F, Vitor-Silva S, Sampaio V, Mendes M, Souza B, et al. Quantitative G6PD Deficiency Screening in Routine Malaria Diagnostic Units in the Brazilian Amazon (SAFEPRIM): An Operational Mixed-Methods Study. Pathogens. 2022;11(11):1328. doi: 10.3390/pathogens11111328 36422580 PMC9696723

[29] Brito-Sousa JD, Peixoto HM, Devine A, Silva-Neto AV, Balieiro PCS, Sampaio VS, et al. Real-life quantitative G6PD screening in Plasmodium vivax patients in the Brazilian Amazon: A cost-effectiveness analysis. PLoS Negl Trop Dis. 2022;16(3):e0010325. doi: 10.1371/journal.pntd.0010325 35324892 PMC8982881

[30] Brito M, Rufatto R, Brito-Sousa JD, Murta F, Sampaio V, Balieiro P, et al. Operational effectiveness of tafenoquine and primaquine for the prevention of Plasmodium vivax recurrence in Brazil: a retrospective observational study. Lancet Infect Dis. 2024;24(6):629–38. doi: 10.1016/S1473-3099(24)00074-4 38452779 PMC7615970

[31] Nascimento JR, Brito-Sousa JD, Almeida ACG, Melo MM, Costa MRF, Barbosa LRA, et al. Prevalence of glucose 6-phosphate dehydrogenase deficiency in highly malaria-endemic municipalities in the Brazilian Amazon: A region-wide screening study. Lancet Reg Health Am. 2022;12:100273. doi: 10.1016/j.lana.2022.100273 36776424 PMC9903920

[32] Brito M, Rufatto R, Murta F, Sampaio V, Balieiro P, Baía-Silva D, et al. Operational feasibility of Plasmodium vivax radical cure with tafenoquine or primaquine following point-of-care, quantitative glucose-6-phosphate dehydrogenase testing in the Brazilian Amazon: a real-life retrospective analysis. Lancet Glob Health. 2024;12(3):e467–77. doi: 10.1016/S2214-109X(23)00542-9 38365417 PMC10882209

[33] Wu Y-H, Tseng C-P, Cheng M-L, Ho H-Y, Shih S-R, Chiu DT-Y. Glucose-6-Phosphate Dehydrogenase Deficiency Enhances Human Coronavirus 229E Infection. The Journal of Infectious Diseases. 2008;197(6):812–6. doi: 10.1086/528377 18269318 PMC7199897

[34] Cappellini M, Fiorelli G. Glucose-6-phosphate dehydrogenase deficiency. The Lancet. 2008;371(9606):64–74. doi: 10.1016/s0140-6736(08)60073-2 18177777

[35] Jain SK, Parsanathan R, Levine SN, Bocchini JA, Holick MF, Vanchiere JA. The potential link between inherited G6PD deficiency, oxidative stress, and vitamin D deficiency and the racial inequities in mortality associated with COVID-19. Free Radic Biol Med. 2020;161:84–91. doi: 10.1016/j.freeradbiomed.2020.10.002 33038530 PMC7539020

[36] Yang H-C, Ma T-H, Tjong W-Y, Stern A, Chiu DT-Y. G6PD deficiency, redox homeostasis, and viral infections: implications for SARS-CoV-2 (COVID-19). Free Radical Research. 2021;55(4):364–74. doi: 10.1080/10715762.2020.1866757 33401987 PMC7799378

[37] Galvão MHR, Roncalli AG. Factors associated with increased risk of death from covid-19: a survival analysis based on confirmed cases. Rev Bras Epidemiol. 2021;23:e200106. doi: 10.1590/1980-549720200106 33439939

[38] Maciel JAC, Pará JW de S, Monteiro AKA, Araújo FE dos S, Siqueira JC de, Sousa JR, et al. Análise da evolução espacial e fatores associados à morbimortalidade por Covid-19 nas regiões geográficas do Brasil: um estudo ecológico. Cad saúde colet. 2023;31(3). doi: 10.1590/1414-462x202331030512

[39] Williamson EJ, Walker AJ, Bhaskaran K, Bacon S, Bates C, Morton CE, et al. Factors associated with COVID-19-related death using OpenSAFELY. Nature. 2020;584(7821):430–6. doi: 10.1038/s41586-020-2521-4 32640463 PMC7611074

[40] Zhou F, Yu T, Du R, Fan G, Liu Y, Liu Z, et al. Clinical course and risk factors for mortality of adult inpatients with COVID-19 in Wuhan, China: a retrospective cohort study. Lancet. 2020;395(10229):1054–62. doi: 10.1016/S0140-6736(20)30566-3 32171076 PMC7270627

[41] Sze S, Pan D, Nevill CR, Gray LJ, Martin CA, Nazareth J, et al. Ethnicity and clinical outcomes in COVID-19: A systematic review and meta-analysis. EClinicalMedicine. 2020;29:100630. doi: 10.1016/j.eclinm.2020.100630 33200120 PMC7658622

[42] Baqui P, Bica I, Marra V, Ercole A, van der Schaar M. Ethnic and regional variations in hospital mortality from COVID-19 in Brazil: a cross-sectional observational study. Lancet Glob Health. 2020;8(8):e1018–26. doi: 10.1016/S2214-109X(20)30285-0 32622400 PMC7332269

[43] Yu Z, Xiong Q, Wang Z, Li L, Niu T. Global, regional, and national burden of glucose-6-phosphate dehydrogenase (G6PD) deficiency from 1990 to 2021: a systematic analysis of the global burden of disease study 2021. Front Genet. 2025;16:1593728. doi: 10.3389/fgene.2025.1593728 40486677 PMC12141281

[44] Frank JE. Diagnosis and management of G6PD deficiency. Am Fam Physician. 2005;72(7):1277–82. 16225031

[45] De Angelis M, Amatore D, Checconi P, Zevini A, Fraternale A, Magnani M, et al. Influenza Virus Down-Modulates G6PD Expression and Activity to Induce Oxidative Stress and Promote Its Replication. Front Cell Infect Microbiol. 2022;11:804976. doi: 10.3389/fcimb.2021.804976 35071051 PMC8770543

[46] Ley B, Alam MS, Satyagraha AW, Phru CS, Thriemer K, Tadesse D, et al. Variation in Glucose-6-Phosphate Dehydrogenase activity following acute malaria. PLoS Negl Trop Dis. 2022;16(5):e0010406. doi: 10.1371/journal.pntd.0010406 PMC9094517 35544453

