# Supplementary material for: Is glucose-6-phosphate dehydrogenase deficiency associated with COVID-19 infection, severity, and death? A cohort study from the Brazilian Amazon
Source: PLoS One. 2025 Dec 23;20(12):e0331729. doi: 10.1371/journal.pone.0331729 (PMC12725547; doi:10.1371/journal.pone.0331729)
Supplement: S1 Table — Only participants who had been screened for infectious diseases at the time of G6PD testing were included. (DOCX) [file pone.0331729.s001.docx]

**S1. Table**: Descriptive and regression sensitivity analysis of COVID-19 incidence in individuals with and without G6PD deficiency. Only participants who had been screened for infectious diseases at the time of G6PD testing were included.

|  | **Descriptive** | | | | **Univariate Regression** | | | **Multivariate Regression** | | |
| --- | --- | --- | --- | --- | --- | --- | --- | --- | --- | --- |
| **Characteristic** | **Total**  N = 2,484 | **No Covid**  N = 2,199 | **With Covid**  N = 285 | **p-value^1^** | **OR^2^** | **95% CI^2^** | **p-value** | **OR^2^** | **95% CI^2^** | **p-value** |
| **G6PD deficient, N (%)** | 38 (1.53%) | 30 (1.36%) | 8 (2.81%) | 0.071 | 2.09 | 0.88; 4.39 | 0.068 | 2.17 | 0.91; 4.61 | 0.057 |
| **Age, mean (SD)** | 34.5 (17.0) | 33.9 (17.1) | 39.3 (15.3) | **<0.001** | 1.02 | 1.01; 1.03 | **<0.001** | 1.02 | 1.01; 1.03 | **<0.001** |
| **Race, N (%)** |  |  |  | **<0.001** |  |  |  |  |  |  |
| White | 209 (8.41%) | 188 (8.55%) | 21 (7.37%) |  | — | — |  | — | — |  |
| Black | 571 (22.99%) | 513 (23.33%) | 58 (20.35%) |  | 1.01 | 0.61; 1.75 | >0.9 | 0.93 | 0.55; 1.61 | 0.8 |
| Asian | 111 (4.47%) | 85 (3.87%) | 26 (9.12%) |  | 2.74 | 1.46; 5.18 | **0.002** | 2.67 | 1.42; 5.09 | **0.002** |
| Brown | 1,555 (62.60%) | 1,384 (62.94%) | 171 (60.00%) |  | 1.11 | 0.70; 1.83 | 0.7 | 1.12 | 0.71; 1.87 | 0.6 |
| Indigenous | 38 (1.53%) | 29 (1.32%) | 9 (3.16%) |  | 2.78 | 1.12; 6.52 | **0.022** | 2.76 | 1.10; 6.50 | **0.023** |
| ^1^Fisher's exact test; Wilcoxon rank sum test | | | | | | | | | | |
| ^2^OR = Odds Ratio, CI = Confidence Interval | | | | | | | | | | |
