# Supplementary material for: Is glucose-6-phosphate dehydrogenase deficiency associated with COVID-19 infection, severity, and death? A cohort study from the Brazilian Amazon
Source: PLoS One. 2025 Dec 23;20(12):e0331729. doi: 10.1371/journal.pone.0331729 (PMC12725547; doi:10.1371/journal.pone.0331729)
Supplement: S2 Table — Only participants who had been screened for infectious diseases at the time of G6PD testing were included. (DOCX) [file pone.0331729.s002.docx]

**S2. Table:** Descriptive and regression sensitivity analysis of COVID-19 hospitalization in individuals with and without G6PD deficiency. Only participants who had been screened for infectious diseases at the time of G6PD testing were included.

|  | **Descriptive** | | | | **Univariate Regression** | | | **Multivariate Regression** | | |
| --- | --- | --- | --- | --- | --- | --- | --- | --- | --- | --- |
| **Characteristic** | **Total**  N = 2,484 | **Not hospitalized**  N = 2.448 | **Interned for Covid**  N = 36 | **p-value^1^** | **OR^2^** | **95% CI^2^** | **p-value** | **OR^2^** | **95% CI^2^** | **p-value** |
| **G6PD deficient, N (%)** | 38 (1.53%) | 37 (1.51%) | 1 (2.78%) | 0.4 | 1.86 | 0.10; 8.99 | 0.5 | 1.71 | 0.09; 8.93 | 0.6 |
| **Age, mean (SD)** | 34.5 (17.0) | 34.2 (16.8) | 54.7 (17.7) | **<0.001** | 1.07 | 1.05; 1.10 | **<0.001** | 1.07 | 1.05; 1.10 | **<0.001** |
| **Race, N (%)** |  |  |  | 0.7 |  |  |  |  |  |  |
| White | 209 (8.41%) | 205 (8.37%) | 4 (11.11%) |  | — | — |  | — | — |  |
| Black | 571 (22.99%) | 563 (23.00%) | 8 (22.22%) |  | 0.73 | 0.23; 2.75 | 0.6 | 0.51 | 0.15; 1.99 | 0.3 |
| Asian | 111 (4.47%) | 109 (4.45%) | 2 (5.56%) |  | 0.94 | 0.13; 4.90 | >0.9 | 0.82 | 0.11; 4.44 | 0.8 |
| Brown | 1,555 (62.60%) | 1,534 (62.66%) | 21 (58.33%) |  | 0.70 | 0.26; 2.42 | 0.5 | 0.72 | 0.26; 2.56 | 0.6 |
| Indigenous | 38 (1.53%) | 37 (1.51%) | 1 (2.78%) |  | 1.39 | 0.07; 9.69 | 0.8 | 1.91 | 0.09; 14.1 | 0.6 |
| ^1^Fisher's exact test; Wilcoxon rank sum test | | | | | | | | | | |
| ^2^OR = Odds Ratio, CI = Confidence Interval | | | | | | | | | | |
