# Supplementary material for: Is glucose-6-phosphate dehydrogenase deficiency associated with COVID-19 infection, severity, and death? A cohort study from the Brazilian Amazon
Source: PLoS One. 2025 Dec 23;20(12):e0331729. doi: 10.1371/journal.pone.0331729 (PMC12725547; doi:10.1371/journal.pone.0331729)
Supplement: S3 Table — Only participants who had been screened for infectious diseases at the time of G6PD testing were included. (DOCX) [file pone.0331729.s003.docx]

**S3. Table:** Descriptive and regression sensitivity analysis of death from COVID-19 in individuals with and without G6PD deficiency. Only participants who had been screened for infectious diseases at the time of G6PD testing were included.

|  | **Descriptive** | | | | **Univariate Regression** | | | **Multivariate Regression** | | |
| --- | --- | --- | --- | --- | --- | --- | --- | --- | --- | --- |
| **Characteristic** | **Total**  N = 2,484 | **No death**  N = 2.468 | **After Covid** N = 16 | **p-value^1^** | **OR^2^** | **95% CI^2^** | **p-value** | **OR^2^** | **95% CI^2^** | **p-value** |
| **G6PD deficient, N (%)** | 38 (1.53%) | 38 (1.54%) | 0 (0.00%) | >0.9 | 0.00 |  | >0.9 | 0.00 |  | >0.9 |
| **Age, mean (SD)** | 34.5 (17.0) | 34.4 (16.9) | 52.0 (17.4) | **<0.001** | 1.06 | 1.03, 1.09 | **<0.001** | 1.06 | 1.03; 1.10 | **<0.001** |
| **Race, N (%)** |  |  |  | 0.2 |  |  |  |  |  |  |
| White | 209 (8.41%) | 209 (8.47%) | 0 (0.00%) |  | — | — |  | — | — |  |
| Black | 571 (22.99%) | 565 (22.89%) | 6 (37.50%) |  | Inf^4^ | 0.00; NA^3^ | >0.9 | Inf^4^ | 0.00; NA^3^ | >0.9 |
| Asian | 111 (4.47%) | 111 (4.50%) | 0 (0.00%) |  | 1.00 | 0.00; Inf^4^ | >0.9 | 0.83 | 0.00; Inf^4^ | >0.9 |
| Brown | 1,555 (62.60%) | 1,546 (62.64%) | 9 (56.25%) |  | Inf^4^ | 0.00; NA^3^ | >0.9 | Inf^4^ | 0.00; NA^3^ | >0.9 |
| Indigenous | 38 (1.53%) | 37 (1.50%) | 1 (6.25%) |  | Inf^4^ | 0.00; NA^3^ | >0.9 | Inf^4^ | 0.00; NA^3^ | >0.9 |
| ^1^Fisher's exact test; Wilcoxon rank sum test | | | | | | | | | | |
| ^2^OR = Odds Ratio, CI = Confidence Interval  ^3^NA= Not Applicable  ^4^Inf= a very large numeric value | | | | | | | | | | |
