# Supplementary material for: Is glucose-6-phosphate dehydrogenase deficiency associated with COVID-19 infection, severity, and death? A cohort study from the Brazilian Amazon
Source: PLoS One. 2025 Dec 23;20(12):e0331729. doi: 10.1371/journal.pone.0331729 (PMC12725547; doi:10.1371/journal.pone.0331729)
Supplement: S4 Table — (DOCX) [file pone.0331729.s004.docx]

**S4. Table:** Descriptive and regression sensitivity analysis of COVID-19 infection in a subsample matched by age.

|  | **Descriptive** | | | | **Univariate Regression** | | | **Multivariate Regression** | | |
| --- | --- | --- | --- | --- | --- | --- | --- | --- | --- | --- |
| **Characteristic** | **Total** | **No Covid**  N = 377 | **With Covid**  N = 35 | **p-value^1^** | **OR^2^** | **95% CI^2^** | **p-value** | **OR^2^** | **95% CI^2^** | **p-value** |
| **G6PD deficient, N (%)** | 206 (50.00%) | 185 (49.07%) | 21 (60.00%) | 0.2 | 1.56 | 0.78; 3.22 | 0.2 | 1.72 | 0.84; 3.66 | 0.15 |
| **Age, mean (SD)** | 33.5 (18.4) | 33.1 (18.5) | 37.5 (16.3) | 0.2 | 1.01 | 0.99; 1.03 | 0.2 | 1.01 | 0.99; 1.03 | 0.2 |
| **Race, N (%)** |  |  |  | 0.2 |  |  |  |  |  |  |
| White | 24 (5.83%) | 22 (5.84%) | 2 (5.71%) |  | — | — |  | — | — |  |
| Black | 24 (5.83%) | 22 (5.84%) | 2 (5.71%) |  | 1.00 | 0.11; 8.95 | >0.9 | 0.79 | 0.09; 7.24 | 0.8 |
| Asian | 14 (3.40%) | 11 (2.92%) | 3 (8.57%) |  | 3.00 | 0.44; 25.4 | 0.3 | 3.32 | 0.46; 29.2 | 0.2 |
| Brown | 345 (83.74%) | 318 (84.35%) | 27 (77.14%) |  | 0.93 | 0.26; 6.02 | >0.9 | 0.91 | 0.25; 5.95 | >0.9 |
| Indigenous | 5 (1.21%) | 4 (1.06%) | 1 (2.86%) |  | 2.75 | 0.11; 36.6 | 0.5 | 2.83 | 0.11; 38.0 | 0.4 |
| ^1^Pearson's Chi-squared test; Wilcoxon rank sum test; Fisher's exact test | | | | | | | | | | |
| ^2^OR = Odds Ratio, CI = Confidence Interval | | | | | | | | | | |
