# Supplementary material for: Is glucose-6-phosphate dehydrogenase deficiency associated with COVID-19 infection, severity, and death? A cohort study from the Brazilian Amazon
Source: PLoS One. 2025 Dec 23;20(12):e0331729. doi: 10.1371/journal.pone.0331729 (PMC12725547; doi:10.1371/journal.pone.0331729)
Supplement: S5 Table — (DOCX) [file pone.0331729.s005.docx]

**S5. Table:** Descriptive and regression sensitivity analysis of hospitalization due to COVID-19 in a sample matched by age.

|  | **Descriptive** | | | | **Univariate Regression** | | | **Multivariate Regression** | | |
| --- | --- | --- | --- | --- | --- | --- | --- | --- | --- | --- |
| **Characteristic** | **Total** | **Not hospitalized**  N = 405 | **Hospitalized for Covid**  N = 7 | **p-value^1^** | **OR^2^** | **95% CI^2^** | **p-value** | **OR^2^** | **95% CI^2^** | **p-value** |
| **G6PD deficient, N (%)** | 206 (50.00%) | 203 (50.12%) | 3 (42.86%) | >0.9 | 0.75 | 0,15; 3.4 | 0.7 | 1.19 | 0.23; 6.15 | 0.8 |
| **Age, mean (SD)** | 33.5 (18.4) | 33.3 (18.3) | 45.7 (20.1) | 0.14 | 1.0 | 1.0; 1.1 | 0.081 | 1.03 | 0.99; 1.08 | 0.10 |
| **Race, N (%)** |  |  |  | 0.074 |  |  |  |  |  |  |
| White | 24 (5.83%) | 24 (5.93%) | 0 (0.00%) |  | — | — |  | — | — |  |
| Black | 24 (5.83%) | 24 (5.93%) | 0 (0.00%) |  | 1.0 | Inf^4^; Inf^4^ | >0.9 | 0.62 | 0.00; Inf^4^ | >0.9 |
| Asian | 14 (3.40%) | 12 (2.96%) | 2 (28.57%) |  | Inf^4^ | Inf^4^; NA^3^ | >0.9 | Inf^4^ | 0.00; Inf^4^ | >0.9 |
| Brown | 345 (83.74%) | 340 (83.95%) | 5 (71.43%) |  | Inf^4^ | Inf^4^; NA^3^ | >0.9 | Inf^4^ | 0.00; Inf^4^ | >0.9 |
| Indigenous | 5 (1.21%) | 5 (1.23%) | 0 (0.00%) |  | 1.0 | Inf^4^; Inf^4^ | >0.9 | 0.97 | 0.00; Inf^4^ | >0.9 |
| ^1^Fisher's exact test; Wilcoxon rank sum test | | | | | | | | | | |
| ^2^OR = Odds Ratio, CI = Confidence Interval  ^3^NA= Not Applicable  ^4^Inf= a very large numeric value | | | | | | | | | | |
