# Supplementary material for: Is glucose-6-phosphate dehydrogenase deficiency associated with COVID-19 infection, severity, and death? A cohort study from the Brazilian Amazon
Source: PLoS One. 2025 Dec 23;20(12):e0331729. doi: 10.1371/journal.pone.0331729 (PMC12725547; doi:10.1371/journal.pone.0331729)
Supplement: S6 Table — (DOCX) [file pone.0331729.s006.docx]

**S6. Table**: Descriptive and regression sensitivity analysis of death from COVID-19 in a sample matched by age.

|  | **Descriptive** | | | | **Univariate Regression** | | | **Multivariate Regression** | | |
| --- | --- | --- | --- | --- | --- | --- | --- | --- | --- | --- |
| **Characteristic** | **Total**  N = 412 | **Survival**  N = 412 | **Death by Covid**  N = 0 | **p-value^1^** | **OR^2^** | **95% CI^2^** | **p-value** | **OR^2^** | **95% CI^2^** | **p-value** |
| **G6PD deficient, n/N (%)** | 206 (50.00%) | 206 (50.00%) | 0 (NaN^5^%) | >0.9 | 1.00 | 0.00; Inf^4^ | >0.9 | 1.00 | 0.00; Inf^4^ | >0.9 |
| **Age, Average (SD)** | 33.5 (18.4) | 33.5 (18.4) | NA^3^ (ON) |  | 1.00 | 0.00; Inf^4^ | >0.9 | 1.00 | 0.00; Inf^4^ | >0.9 |
| **Race, n/N (%)** |  |  |  | >0.9 |  |  |  |  |  |  |
| White | 24 (5.83%) | 24 (5.83%) | 0 (NaN^5^%) |  | — | — |  | — | — |  |
| Black | 24 (5.83%) | 24 (5.83%) | 0 (NaN^5^%) |  | 1.00 | 0.00; Inf^4^ | >0.9 | 1.00 | 0.00; Inf^4^ | >0.9 |
| Asian | 14 (3.40%) | 14 (3.40%) | 0 (NaN^5^%) |  | 1.00 | 0.00; Inf^4^ | >0.9 | 1.00 | 0.00; Inf^4^ | >0.9 |
| Brown | 345 (83.74%) | 345 (83.74%) | 0 (NaN^5^%) |  | 1.00 | 0.00; Inf^4^ | >0.9 | 1.00 | 0.00; Inf^4^ | >0.9 |
| Indigenous | 5 (1.21%) | 5 (1.21%) | 0 (NaN^5^%) |  | 1.00 | 0.00; Inf^4^ | >0.9 | 1.00 | 0.00; Inf^4^ | >0.9 |
| ^1^Fisher's exact test | | | | | | | | | | |
| ^2^OR = Odds Ratio, CI = Confidence Interval  ^3^NA= Not Applicable  ^4^Inf= a very large numeric value  ^5^NaN= undefined | | | | | | | | | | |
